# A tuberculosis nationwide prevalence survey in Gambia, 2012

**DOI:** 10.2471/BLT.14.151670

**Published:** 2016-04-21

**Authors:** Ifedayo MO Adetifa, Lindsay Kendall, Adedapo Bashorun, Christopher Linda, Semeeh Omoleke, David Jeffries, Rahmatulai Maane, Beatrice Dei Alorse, William Dei Alorse, Catherine Bi Okoi, Kodjovi D Mlaga, Ma Ansu Kinteh, Simon Donkor, Bouke C de Jong, Martin Antonio, Umberto d’Alessandro

**Affiliations:** a Department of Infectious Diseases Epidemiology, London School of Hygiene & Tropical Medicine, Keppel Street, London, WC1E 7HT, England.; b Disease Control and Elimination Theme, Medical Research Council Unit, Banjul, Gambia.; c Clinical Services Department, Medical Research Council Unit, Banjul, Gambia.; d Vaccinology Theme, Medical Research Council Unit, Banjul, Gambia.; e Mycobacteriology Unit, Institute of Tropical Medicine, Antwerp, Belgium.

## Abstract

**Objective:**

To estimate the population prevalence of active pulmonary tuberculosis in Gambia.

**Methods:**

Between December 2011 and January 2013, people aged ≥ 15 years participating in a nationwide, multistage cluster survey were screened for active pulmonary tuberculosis with chest radiography and for tuberculosis symptoms. For diagnostic confirmation, sputum samples were collected from those whose screening were positive and subjected to fluorescence microscopy and liquid tuberculosis cultures. Multiple imputation and inverse probability weighting were used to estimate tuberculosis prevalence.

**Findings:**

Of 100 678 people enumerated, 55 832 were eligible to participate and 43 100 (77.2%) of those participated. A majority of participants (42 942; 99.6%) were successfully screened for symptoms and by chest X-ray. Only 5948 (13.8%) were eligible for sputum examination, yielding 43 bacteriologically confirmed, 28 definite smear-positive and six probable smear-positive tuberculosis cases. Chest X-ray identified more tuberculosis cases (58/69) than did symptoms alone (43/71). The estimated prevalence of smear-positive and bacteriologically confirmed pulmonary tuberculosis were 90 (95% confidence interval, CI: 53–127) and 212 (95% CI: 152–272) per 100 000 population, respectively. Tuberculosis prevalence was higher in males (333; 95% CI: 233–433) and in the 35–54 year age group (355; 95% CI: 219–490).

**Conclusion:**

The burden of tuberculosis remains high in Gambia but lower than earlier estimates of 490 per 100 000 population in 2010. Less than half of all cases would have been identified based on smear microscopy results alone. Successful control efforts will require interventions targeting men, increased access to radiography and more accurate, rapid diagnostic tests.

## Résumé

**Objectif:**

Estimer la prévalence de la tuberculose pulmonaire active dans la population de Gambie.

**Méthodes:**

Entre décembre 2011 et janvier 2013, dans le cadre d'une enquête nationale en grappes à plusieurs degrés, un dépistage de la tuberculose pulmonaire active a été réalisé au moyen d'un questionnaire sur les symptômes tuberculeux et d'une radiographie pulmonaire, chez des personnes âgées de 15 ans ou plus ayant consenti à participer. Pour confirmer le diagnostic, des échantillons d'expectoration ont été collectés pour tous les participants dont les résultats de dépistage s’étaient révélés positifs, en vue de réaliser des examens par microscopie de fluorescence et des cultures en milieu liquide. Les techniques d'imputation multiple et de pondération par l'inverse de la probabilité ont été utilisées pour estimer la prévalence de la tuberculose.

**Résultats:**

Sur les 100 678 personnes recensées, 55 832 étaient éligibles pour participer à l'étude et, parmi elles, 43 100 personnes (77,2%) ont participé. La majorité des participants (42 942; 99,6%) ont effectivement répondu au questionnaire sur les symptômes et passé une radiographie pulmonaire. Seules 5 948 personnes (13,8%) ont été éligibles pour un examen des expectorations, ce qui a entraîné le dépistage de 43 cas de tuberculose confirmée bactériologiquement, de 28 cas formels de tuberculose à frottis positif et de 6 cas probables de tuberculose à frottis positif. Les radiographies pulmonaires ont permis d'identifier plus de cas de tuberculose (58/69) que le seul questionnaire sur les symptômes (43/71). Les prévalences de la tuberculose pulmonaire à frottis positif et de la tuberculose bactériologiquement confirmée ont respectivement été estimées à 90 cas (intervalle de confiance de 95%: 53–127) pour 100 000 habitants et 212 cas (IC 95%: 152–272) pour 100 000 habitants. La prévalence de la tuberculose s'est avérée plus élevée dans la population masculine (333 cas; IC 95%: 233–433) et dans la tranche des 35–54 ans (355; IC 95%: 219-490).

**Conclusion:**

La charge de la tuberculose reste élevée en Gambie, même si elle a baissé par rapport à la précédente estimation, réalisée en 2010, qui faisait état de 490 cas pour 100 000 habitants. En utilisant uniquement la microscopie des frottis, moins de la moitié des cas auraient été identifiés. Pour être efficaces, les efforts de lutte contre la tuberculose doivent inclure des campagnes ciblant les hommes, améliorer l'accessibilité des examens radiographiques et utiliser des tests diagnostiques rapides plus précis.

## Resumen

**Objetivo:**

Estimar la prevalencia de la tuberculosis pulmonar activa entre la población en Gambia.

**Métodos:**

Entre diciembre de 2011 y enero de 2013, personas de 15 años o más que participaron en una encuesta nacional en varias etapas y a nivel de grupos fueron examinadas en busca de tuberculosis pulmonar activa mediante una radiografía de tórax y para encontrar síntomas de tuberculosis. Para confirmar el diagnóstico, se recolectaron muestras de esputo de las personas cuyo examen resultó positivo y se sometieron a microscopia de fluorescencia y cultivos líquidos de tuberculosis. Para calcular la prevalencia de la tuberculosis, se utilizaron varias asignaciones y análisis de probabilidad inversa.

**Resultados:**

De las 100 678 personas enumeradas, 55 832 se consideraron aptas para participar, de las cuales 43 100 (77,2%) participaron. La mayoría de los participantes (42 942; 99,6%) fueron examinados con éxito para detectar síntomas, así como por radiografía del tórax. Solo 5 948 (13,8%) fueron aptos para pruebas de esputo, lo que dio lugar a 43 confirmados a nivel bacteriológico, 28 casos bacilíferos de tuberculosis definitivos y 6 casos bacilíferos de tuberculosis probable. Las radiografías del tórax identificaron más casos de tuberculosis (58/69) que los síntomas por sí solos (43/71). La prevalencia estimada de tuberculosis pulmonar bacilífera y confirmada a nivel bacteriológico fue de 90 (intervalo de confianza, IC, del 95%: 53–127) y 212 (IC del 95%: 152–272) por cada 100 000 habitantes, respectivamente. La prevalencia de la tuberculosis fue mayor en hombres (333; IC del 95%: 233–433) y en los grupos de edad entre 35 y 54 años (355; IC del 95%: 219–490).

**Conclusión:**

Los índices de tuberculosis siguen siendo altos en Gambia, pero inferiores a las estimaciones anteriores de 490 por cada 100 000 habitantes en 2010. Se identificaron menos de la mitad de todos los casos según los resultados de la microscopia de frotis por sí solos. Para que los esfuerzos de control tengan éxito, serán necesarias intervenciones enfocadas a los hombres, un mayor acceso a radiografías y pruebas de diagnóstico más rápidas y exactas.

## ملخص

**الغرض:**

تقدير مدى تفشي مرض السل الرئوي النشط بين السكان في غامبيا.

**الطريقة:**

خضع الأشخاص الذين تبلغ أعمارهم 15 عامًا أو أكثر ممن شاركوا في مسح عنقودي متعدد المراحل على مستوى الدولة بأكملها في الفترة بين ديسمبر/كانون الأول 2011 ويناير 2013 لفحص للكشف عن الإصابة بالسل الرئوي النشط باستخدام تصوير الصدر بالأشعة وفحص للكشف عن أعراض السل. وللتأكد من التشخيص، تم جمع عينات من البصاق من الأشخاص الذين أظهر الفحص نتائج إيجابية لهم، وخضعت تلك العينات للفحص تحت المجهر الفلوري ولعمليات زراعة في وسط سائل للكشف عن بكتريا السل. وتمت الاستعانة بطريقة حساب القيم التعويضية المتعددة وأسلوب ترجيح الاحتمال العكسي في تقدير مدى تفشي مرض السل.

ا **لنتائج ** بلغ عدد الأشخاص المؤهلين للمشاركة 55,832 شخص من بين الأشخاص الذين تم إحصاء أعدادهم والتي بلغت 100,678، وقد شارك منهم بالفعل 43,100 شخص (بنسبة 77.2‏%). وخضع معظم المشاركين (42942؛ بنسبة 99.6‏%) لإجراء ناجح للفحص الذي يكشف عن ظهور أعراض المرض وذلك الذي يعتمد على تصوير الصدر بالأشعة السينية. وبلغ عدد المؤهلين لاختبار البصاق 5948 شخصًا فقط (بنسبة 13.8‏%)، حيث ظهر من بين هؤلاء الأشخاص حالات للإصابة بالسل تم تأكيد تشخيصها بالطرق البكتريولوجية لدى 43 شخصًا، وتأكدت النتائج الإيجابية لفحص المسحة لدى 28 شخصًا، وأظهر ستة أشخاص نتائج إيجابية محتملة لفحص المسحة تشير للإصابة بالمرض. وتم تحديد المزيد من حالات الإصابة بالسل (58/69) من خلال تصوير الصدر بالأشعة السينية مقارنةً باستخدام الكشف عن ظهور الأعراض فقط (43/71). ووفقًا للتقديرات، فإن تفشي حالات الإصابة بالسل الرئوي التي تم تأكيد تشخيصها من خلال النتائج الإيجابية لفحص مسحة البصاق والطرق البكتريولوجية بلغ معدلًا مقداره 90 حالة (بنسبة أرجحية مقدارها 95‏%: 53 – 127) و212 حالة (بنسبة أرجحية مقدارها 95‏%: 152 – 272) في كل مجموعة سكانية يبلغ عدد أفرادها 100,000 نسمة، على التوالي. وارتفعت نسبة تفشي مرض السل عند الذكور (333؛ بنسبة أرجحية مقدارها 95‏%: 233 – 433) وكذلك في المجموعة العمرية من سن 35 إلى 54 عامًا (355؛ بنسبة أرجحية مقدارها 95‏%: 219–490).

**الاستنتاج:**

يظل عبء مرض السل بمعدل مرتفع في غامبيا، إلا أنه يقل عن التقديرات السابقة التي أشارت إلى وجود 490 حالة في كل مجموعة سكانية يبلغ عدد أفرادها 100,000 نسمة في عام 2010. وقد تم تحديد ما يقل عن النصف من الحالات بأكملها اعتمادًا على نتائج الفحص المجهري لمسحة البصاق وحده. وستتطلب الجهود الرامية للنجاح في السيطرة على المرض بعض التدخلات التي تستهدف الرجال، وزيادة فرص الخضوع للتصوير بالأشعة وتوفير المزيد من الاختبارات التشخيصية الدقيقة والسريعة.

## 摘要

**目的:**

旨在预测冈比亚境内活动性肺结核患病率。

**方法:**

在 2011 年 12 月到 2013 年 1 月期间，研究人员采用 X 线胸片对年龄为 15 岁以及上参与全国范围内、多阶段整群调查的人员进行活动性肺结核筛查以及肺结核症状筛查。为进行确诊，研究人员采集了筛查结果阳性人员的痰液样本并对其进行荧光显微镜观察以及肺结核液体培养基培养。还采用多重填补法和逆概率加权法以估算肺结核患病率。

**结果:**

在选出的 100,678 人中，55 ,832 人有资格参与调查，其中 43,100 (77.2%) 人参与了调查。我们通过 X 线胸片成功地对大部分参与者（42,942 人；99.6%）进行了症状筛查。仅 5,948 (13.8%) 人有资格参加痰液检验，结果显示 43 人为细菌学检查阳性，28 人痰涂片检查为阳性，并且发现了六例痰涂片检查疑似阳性的肺结核病例。相对于单纯症状查痰法 (43/71)， X 线胸片可确诊更多肺结核病例 (58/69)。每 10 万人中，预计痰涂片阳性和细菌学检查阳性的肺结核患病人数分别为 90（95% 置信区间，CI: 53-127) 和 212 (95% 置信区间，CI: 152–272)。男性 (333; 95% CI: 233–433) 以及年龄介于 35–54 岁的人群 (355; 95% CI: 219–490) 肺结核患病率更高 。

**结论:**

冈比亚境内肺结核患病率居高不下，但低于 2010 年估计的每 10 万人中 490 例的患病率。在所有病例中，半数以下病例本可以仅仅通过显微镜观察痰涂片确诊。为有效控制疾病发展，需要对男性采取干预措施、进一步促进 X线胸片以及更多精确、快速诊断检测方法的普及。

## Резюме

**Цель:**

Оценить популяционную распространенность активной формы туберкулеза легких в Гамбии.

**Методы:**

В период с декабря 2011 года по январь 2013 года лица в возрасте ≥□15 лет принимали участие в национальном многоэтапном исследовании с применением гнездовой выборки; их обследовали на наличие активной формы туберкулеза легких при помощи рентгенографии грудной клетки, а также проверяли наличие у них клинических симптомов туберкулеза. С целью подтверждения диагноза у лиц, для которых результаты скринингового обследования были положительными, брали пробу мокроты и подвергали ее флуоресцентной микроскопии, а также применяли ее посев на жидкую питательную среду. Для оценки распространенности туберкулеза применялся метод множественного восстановления пропущенных данных и метод весовых коэффициентов для величин, обратных вероятности.

**Результаты:**

Из 100 678 человек, зарегистрированных для проведения обследования, 55 832 отвечали критериям исследования. Из них 43 100 человек (77,2%) приняли участие в исследовании. В большинстве своем участники (42 942 (99,6%)) успешно прошли скрининг симптомов и рентгенографию грудной клетки. Только 5 948 участников (13,8%) отвечали критериям для сдачи мокроты на анализ, из них в 43 случаях было получено бактериологическое подтверждение, 28 мазков были квалифицированы как достоверно положительные, шесть — как вероятные случаи туберкулеза. Рентгенография грудной клетки выявила больше случаев туберкулеза (58 из 69), чем диагностика только по симптомам (43 из 71). Оценка распространенности положительного мазка на туберкулез и бактериологически подтвержденного туберкулеза легких составила 90 (95%-й доверительный интервал, ДИ: 53–127) и 212 (95%-й ДИ: 152–272) на 100 000 населения соответственно. Туберкулез чаще встречался среди мужчин (333, 95%-й ДИ: 233–433) и лиц в возрасте 35–54 лет (355, 95%-й ДИ: 219–490).

**Вывод:**

В Гамбии сохраняется высокое бремя туберкулеза, но полученные оценочные значения ниже величины, рассчитанной в 2010 году, в 490 случаях на 100 000 населения. Менее половины всех случаев могли быть выявлены только на основании микроскопического исследования мазка. Для успеха контролирующих мероприятий потребуются вмешательства, ориентированные на мужчин, более широкий доступ к рентгенографии и более точные и быстродействующие диагностические тесты.

## Introduction

Tuberculosis killed 1.5 million people in 2014 and is the leading cause of death from an infectious disease worldwide.^1^ Sub-Saharan Africa, with 28% (9.6 million) of all notified tuberculosis cases in 2014, endures a disproportionate burden of the disease relative to its population. In Gambia,‭ the estimated incidence and prevalence of tuberculosis rose from 258 and 350 per 100 000 population respectively in 1990 to 284 and 490 per 100 000 in 2011.^2^ In addition, the tuberculosis case detection rate – that is, the ratio of the number of notified tuberculosis cases to the number of incident tuberculosis cases in a given year – remained low at 48% (95% confidence interval, CI: 40–58).^2^ It is not clear if poor case detection is due to inequitable access to care or inadequate diagnosis of tuberculosis in urban or remote parts of the country.‬‬‬‬‬‬‬‬‬‬‬‬‬‬‬‬‬‬‬

Given the need for improved, evidence-based interventions in tuberculosis control in Gambia, it is important to establish reliable baseline estimates of tuberculosis prevalence against which future control interventions can be assessed. This study therefore aimed to estimate the population prevalence of active pulmonary tuberculosis disease in Gambia, in 2012 and to compare the case detection rate with global tuberculosis control targets.

## Methods

### Study design

We carried out a nationwide, multistage cluster survey in 2011–2013. A sample size of 55 281 participants ≥ 15 years old from 80 clusters was calculated assuming a prevalence of 292 sputum smear-positive cases per 100 000 population,^3^ 85% participation target, design effect of 1.51 and application of a finite population correction.^4^ A sample size with 80 clusters was expected to give around 20% precision and higher than 25% precision under the most plausible scenarios, and with an intracluster coefficient of variation of  0.5 the calculated design effect was 1.51.

Sampling to select survey areas was multistage and without any stratification. First, we allocated 80 survey enumeration areas by regions of the country in proportion to population size based on the national 2003 census (Central Statistics Department, Government of Gambia). Following this allocation, the West Coast region with about 28.7% (389 274) of the population was to contribute 23 enumeration areas and the least populated Lower River region contributed four enumeration areas for 5.3% (72 184) of the population. This procedure was similar in outcome to the recommended sampling in proportion to population size.^4^ Then we randomly selected the survey enumeration areas (e.g. 23 for the West Coast region) up to a total of 80 for the entire country. Each selected survey enumeration area was paired with between one and two adjacent enumeration areas in whole or part until an adult population of 500–700 was attained.

People eligible for participation were all those aged ≥ 15 years; permanent residents who spent at least one night in the household in the preceding 4 weeks; and visitors who had arrived in the household 4 weeks or more before.

### Study procedures

Three teams – each consisting of a research clinician who led a team of 7 trained fieldworkers and a radiographer – performed the fieldwork from December 2011 to January 2013. Data collection in each cluster was done over a seven-day period. The field workers, in collaboration with community-selected liaisons, enumerated the population in each cluster 4–6 weeks before actual data collection. The field workers visited the enumerated households to obtain data on household composition and residency, and at the same time discussed the purpose of the survey. For families that were not at home, the team were informed at which time they could return to meet the household members. Community entry meetings with the local chief and other community leaders were also held to provide further information.

The field operation sites were selected based on input from community members to ensure that location was as central as possible to facilitate access by pedestrian travel. Project vehicles were available for eligible disabled participants to bring them to the site.

Screening of eligible participants followed the World Health Organization (WHO) recommended algorithm for tuberculosis prevalence surveys.^4^ At the field operation site, participants gave written informed consent before they were interviewed for tuberculosis symptoms (cough, weight loss, fever, drenching night sweats, chest pain, shortness of breath, loss of appetite and coughing up of blood). Then they had chest X-rays taken by a radiographer who used a mobile direct digital radiography machine. Chronic cough lasting two weeks or more is the cardinal symptom in the national tuberculosis guidelines. Participants who screened positive for symptoms and/or had an X-ray suggestive of tuberculosis were asked to submit two sputum specimens. All those unable to have chest X-rays were asked to submit two sputum samples whether they had symptoms or not. Participants submitted the sputum samples at the field site, with a 30–45 minute interval. Participants who were unable to submit samples on-site were given two prelabelled containers for collection early the following morning. All survey participants were given a token gift of laundry soap.

### Case ascertainment

Survey clinicians were encouraged to over-interpret X-rays to increase the sensitivity of screening, as recommended.^4^ The radiology panel, which included those who trained the X-ray readers, reviewed all abnormal X-rays and 10% of normal films for quality assurance and definitions. The final radiological diagnosis was determined via consensus by a pulmonologist and radiologist.

Sputum specimens collected in the field were stored in a temperature-monitored cold box. If the samples could not be transported to the laboratory that day, the samples were stored in a fridge kept at 4 C in the team’s camp. In general samples were transported every 48 hours after sputum collection to the main laboratory, the maximum delay in transportation was 72 hours. Sputum samples were processed at the Tuberculosis Diagnostic and Research Laboratory of the Medical Research Council Unit The Gambia. The laboratory holds good clinical laboratory practice^5^ accreditation and subscribes to an external quality assessment service.^6^ Sputum smears were examined using fluorescence microscopy. Cultures were performed using the BACTEC Mycobacterial Growth Indicator Tube (MGIT) system (Becton Dickinson, Franklin Lakes, United States of America). Standard laboratory procedures^7^ were followed for confirmation of growth in liquid culture including rapid species identification with an immunochromatographic assay (MGIT TBc Identification Test, Becton Dickinson, Franklin Lakes, USA). All acid-fast bacillus (AFB) isolates obtained from cultures were classified as either *Mycobacterium tuberculosis* or nontuberculous mycobacteria.

The final survey case classification, as defined in Box 1, was done by a central panel consisting of infectious diseases and chest physicians, epidemiologists, laboratory experts and tuberculosis programme representatives.^4^

Box 1 **Case definitions for the national tuberculosis prevalence survey, Gambia, 2012**

**Laboratory case definitions**

Culture-confirmed tuberculosis case: isolation of *Mycobacterium tuberculosis* complex from a sputum specimen.

Sputum smear-positive tuberculosis case: acid-fast bacillus (AFB)-positive by sputum smear examination, i.e. at least one AFB in 100 immersion fields.

**Definite survey case**

Bacteriologically confirmed tuberculosis case: one positive tuberculosis culture and at least one of the following:

– another sample culture-positive;

– sputum smear-positive; or

– chest X-ray abnormalities suggestive of tuberculosis by central audited reading.

Sputum smear-positive tuberculosis case: one AFB-positive sample and a culture-positive sample.

**Probable smear-positive tuberculosis case**

An AFB-positive sample and at least one of the following:

– AFB-positive in another sample but not culture-positive, and no isolation of nontuberculous mycobacteria; or

– Chest X-ray abnormal at central reading but not culture-positive, and no isolation of nontuberculous mycobacteria.

### Data analysis

There was double entry of survey data in a MySQL version 5.6.19 (Oracle, Redwood Shores, USA) relational database. All data analyses were done using Stata version 12.1 (Stata Corp., College Station, USA). We tested for differences in proportions using two-sample tests of equality of proportions, and conducted multiple imputation to correct for missing data.^8^^,^^9^ For prevalence estimates, we used three modelling approaches with robust standard errors, missing value imputation and inverse probability weighting applied.^4^^,^^8^ With multiple runs of chained imputation data sets, trends for the mean values of the four imputed variables (chest X-ray-positive, AFB-positive, culture-positive and bacteriologically confirmed tuberculosis) were obtained for all iterations. We derived subject-level missing values from the relevant combinations of the imputed variables and stratified all prevalence data by sex, age group and residence (rural/urban). Overall prevalence for all forms of tuberculosis – that is, pulmonary and extra-pulmonary – across all age groups were obtained by calculating pulmonary tuberculosis cases (all ages) as a weighted average of tuberculosis in survey participants and in children (obtained from routine reports, Gambian National Tuberculosis and Leprosy Programme). The value obtained was then revised upwards by the proportion of all notified tuberculosis cases that were the extrapulmonary type. Incidence was calculated based on assumptions from a set of statistical distributions as described elsewhere.^2^

### Ethics approval

The study obtained ethics approval from the joint ethics committee of the Gambian Government and Medical Research Council. All tuberculosis patients identified during the survey were promptly referred to the nearest treatment centre for treatment at no cost and notified to the national tuberculosis programme.

## Results

### Participants

**Fig. 1:**
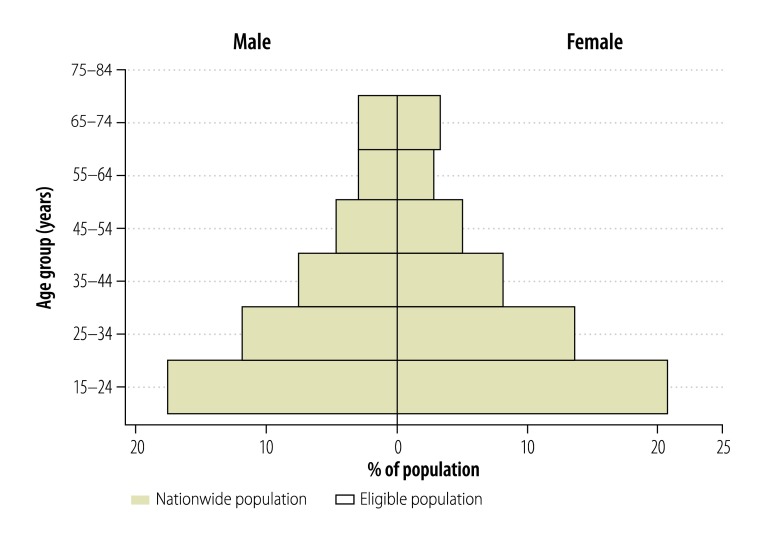
Population pyramid showing representativeness of eligible population in the national tuberculosis prevalence survey, Gambia, 2012

Note: Nationwide population was 1 306 681 and eligible population was 55 832 people.

**Fig. 2:**
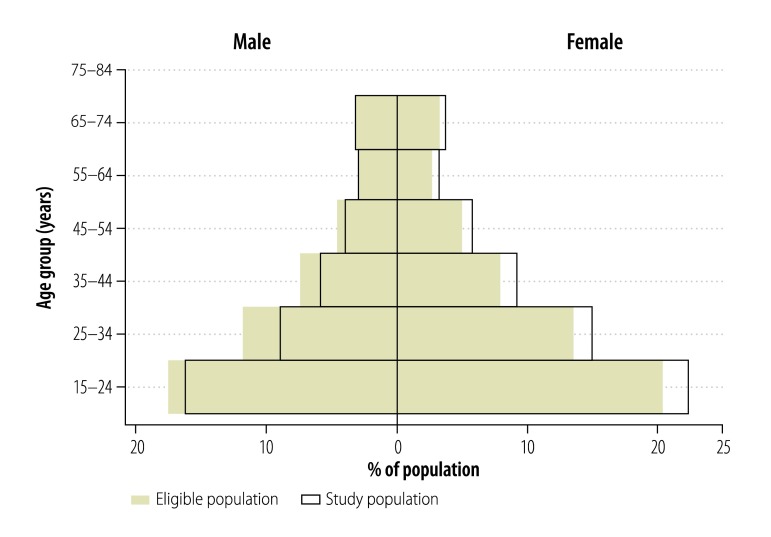
Population pyramid showing representativeness of study population in the national tuberculosis prevalence survey, Gambia, 2012

Note: Eligible population was 55 832 people and study population was 43 100 people.

Enumeration yielded 100 678 people in 13 847 households across 80 clusters, of whom 55 832 (55.5%) were eligible to participate in the survey. The enumerated population was representative of Gambia’s population structure (2003 national census) by age and sex (Fig. 1). Of the 55 832 people invited, 43 100 (77.2%) consented to participate. As shown in Fig. 2, there was a significantly higher proportion of female (84.9%; 25 596/30 153) than male participants (68.2%; 17 504/25 679; *P* < 0.0001). In addition, participation was higher in rural (82.3%; 25 554/31 043) than urban (70.8%; 17 546/24 789) clusters (*P* < 0.0001). Overall, participation was slightly less than the 85% target due to lower participation in urban areas and among males. The median age of participants was 28 years (interquartile range: 20–41 years).

Data source: Gambia 2003 National Census, Central Statistics Department, Government of the Gambia

Data source: Gambia 2003 National Census, Central Statistics Department, Government of the Gambia

### Suspected cases

**Fig. 3:**
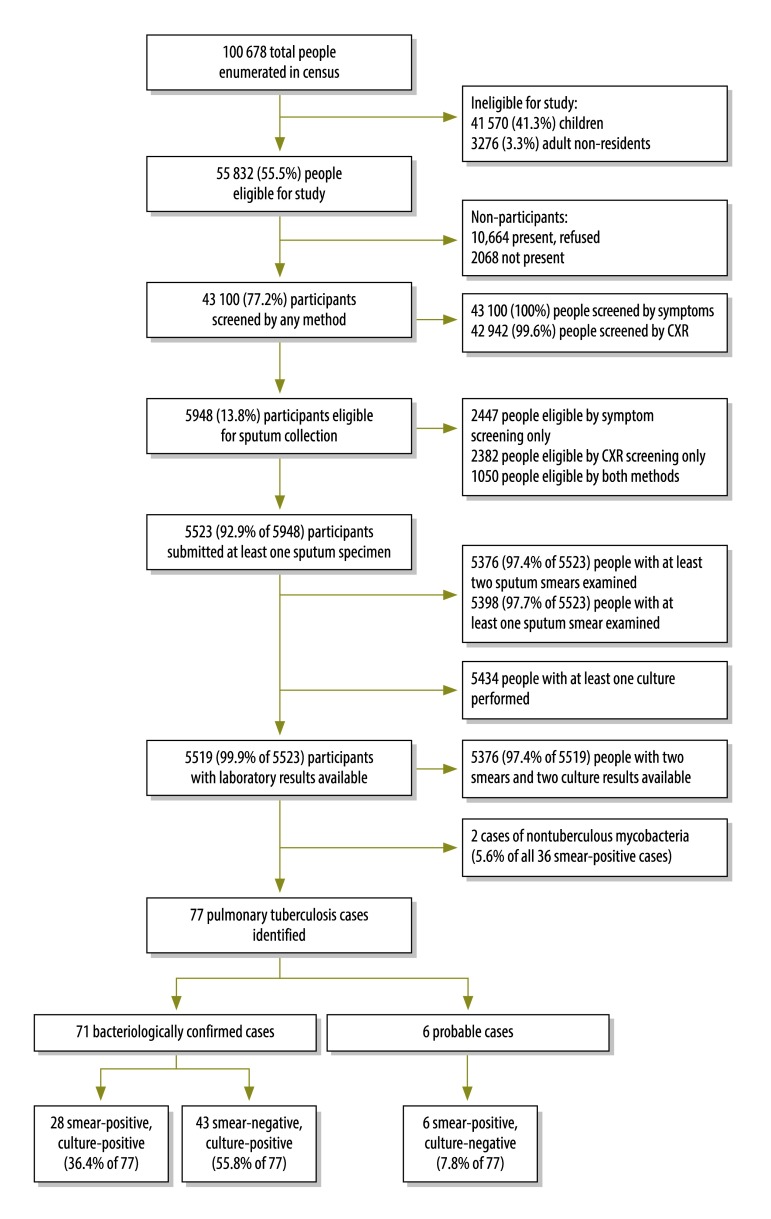
Outline of the national tuberculosis prevalence survey, Gambia, 2012

All participants were successfully screened for symptoms and 42 942 (99.6%) by X-ray (Fig. 3); the majority of missing chest X-rays (145/158, 91.8%) were due to patient refusal or ill health, while the remaining (13/158, 8.2%) were due to technical problems.

CXR: chest X-ray.

Cough was reported by 4802 (11.1%) participants. Of those participants knowing the duration of their cough, 962 (2.2%) had had a cough lasting ≥ 2 weeks, 2979 (6.9%) had coughed for < 1 week and 799 (1.9%) had coughed for 1–2 weeks. With respect to other symptoms in the guidelines, 6637 (15.4%) participants reported fever, 6551 (15.2%) chest pain, 1595 (3.7%) night sweats, 2672 (6.2%) shortness of breath, 3620 (8.4%) anorexia and 8448 (19.6%) weight loss. Using other symptom categories, 1372 (3.2%) participants reported cough of < 2 weeks plus two or more of the other symptoms, while 1128 (2.6%) did not have a cough but had three or more other symptoms.

Based on field X-ray screening, 3407 (7.9%) participants had chest abnormalities suggestive of tuberculosis.

A total of 5948 (13.8%) participants were classified as tuberculosis suspects and were eligible for sputum examination; 2447 were eligible by symptom screening only, 2382 by chest X-ray screening only and 1050 by both screening methods (Fig. 3).

### Definite cases

Laboratory results were available for 5519 participants with suspected tuberculosis. Overall, 77 participants with pulmonary tuberculosis were identified, 71 of which were bacteriologically confirmed. The expert panel categorized 28 participants’ diagnostic results as definite, smear-positive (smear-positive, culture-positive), 43 as definite, bacteriologically confirmed (smear-negative, culture-positive) and six as probable, smear-positive (smear-positive, culture-negative; Fig. 3). Of the samples that were both smear positive and culture positive; 34 samples were identified by culture as *M. tuberculosis* and 2 (5.6%) as nontuberculous mycobacteria.

If sputum smear microscopy had been done only in symptomatic people, diagnosis would have been made in 52.9% (18/34) of prevalent smear-positive cases, while chest X-ray abnormalities would have identified 94.1% (32/34). Among all participants with bacteriologically confirmed tuberculosis, symptoms alone identified significantly fewer cases than chest X-ray screening in the field, 60.6% (43/71) versus 84.1% (58/69; *P* < 0.01), respectively. In addition, 25/77 patients (32.5%) reported cough for ≥ 2 weeks, 14 (18.2%) for < 2 weeks with two or more symptoms, while 5 (6.5%) did not have a cough but had three other symptoms. In total, 24.7% (19/77) of the participants were identified as having tuberculosis because the patient screened positive for additional symptoms.

Tuberculosis was identified predominantly among male participants (51; 66.2%). The 15–34, 35–54 and ≥ 55 year age groups had 37.7% (29), 39.0% (30) and 23.4% (18) of cases respectively. The numbers of tuberculosis cases were similar in urban (39) and rural (38) clusters.

Most participants identified with tuberculosis (94.8%; 73) were not on treatment at the time of the survey and were not officially registered with the national tuberculosis programme. Thirty-eight patients had sought care on account of their symptoms; all 38 had visited health facilities and the great majority of them (31/38) had visited a public health facility.

### Prevalence

There were missing data for smear, culture and symptom identification for 512 participants eligible for sputum examination; 24 of these had missing chest X-rays as well. Bacterial identification was a conditional imputation, conditioned on a positive culture. In addition, 134 subjects had only X-rays missing. In total, 646 subjects required imputation analysis.

For the adjusted prevalence estimates for smear-positive and bacteriologically confirmed pulmonary tuberculosis, the results from the three models were consistent and for models 2 and 3 were quite similar, especially for smear-positive tuberculosis (Table 1). Considering the larger differences for bacteriologically confirmed tuberculosis by residence and sex with model 3, the extrapolation by inverse probability weighting in this model appears to have adjusted for the lower participation in urban areas and by males. This result suggests that model 3 has the best fit for our data. Based on model 3 the estimated prevalence of smear-positive tuberculosis was 90 per 100 000 population (95% CI: 53–127) and for bacteriologically confirmed tuberculosis was 212 per 100 000 (95% CI: 152–272).

**Table 1:** Adjusted overall estimated prevalence of pulmonary tuberculosis per 100 000 population aged ≥ 15 years, Gambia, 2012

Group	Prevalence per 100 000 population (95% CI)					
	Smear-positive cases			Bacteriologically confirmed cases		
	Model 1^a^	Model 2^b^	Model 3^c^	Model 1^a^	Model 2^b^	Model 3^c^
**Overall point estimate**	80 (44–116)	92 (55–128)	90 (53–127)	181 (129–232)	199 (147–250)	212 (152–272)
**Residence**						
Rural	79 (27–132)	90 (44–142)	86 (32–140)	154 (90–219)	165 (102–228)	109 (54–164)
Urban	81 (35–127)	93 (44–142)	96 (43–148)	219 (138–301)	239 (152–327)	266 (164–368)
**Sex**						
Male	139 (82–195)	151 (88–213)	148 (88–208)	295 (208–381)	309 (221–396)	333 (233–433)
Female	40 (0–81)	40 (1–80)	41(0–83)	103 (50–155)	104 (53–156)	109 (54–164)
**Age group (years)**						
15–34	45 (19–71)	53 (23–82)	56 (24–88)	109 (63–155)	117 (70–163)	133 (76–190)
35–54	102 (57–190)	141 (59–224)	144 (65–223)	285 (178–392)	323 (199–447)	355 (219–490)
≥ 55	146 (55–387)	187 (0–385)	159 (0–367)	331 (92–570)	364 (140–588)	329 (99–558)

CI: confidence interval.

^a^ Model 1: logistic regression model with robust standard errors and no missing value imputation.

^b^ Model 2: logistic regression model with robust standard errors and missing value imputation of non-participants as well as participants.

^c^ Model 3: logistic regression model with robust standard errors, with missing value imputation of participants with missing smear and/or culture results, and inverse probability weighting applied to all survey participants to correct for differentials in participation by age, sex and recidence.^4^^,^^8^

Prevalent smear-positive tuberculosis was 3.4-fold higher in male than female participants. Bacteriologically confirmed pulmonary tuberculosis was 2.4-fold higher than smear-positive tuberculosis and 2.4-fold higher in urban than rural areas (Table 1). Table 2 shows age- and sex-associated differences in the burden of smear and bacteriologically confirmed tuberculosis based on estimates from model 3.

**Table 2:** Prevalence of pulmonary tuberculosis by sex and age group per 100 000 population aged ≥ 15 years, Gambia, 2012

Group	Prevalence per 100 000 population (95% CI)^a^	
	Smear-positive cases	Bacteriologically confirmed cases
**15–34 years**		
Male	104 (42–166)	209 (113–305)
Female	33 (0–78)	71 (20–191)
**35–54 years**		
Male	282 (118–445)	634 (384–885)
Female	26 (0–80)	105 (19–191)
**≥ 55 years**		
Male	83 (0–203)	325 (108–543)
Female	227 (0–557)	330 (0–677)

CI: confidence interval.

^a^ Model 3: robust standard errors with missing value imputation and inverse probability weighting; confidence intervals were calculated with exact binomial probability theory.

### Adjusted prevalence

We calculated the adjusted tuberculosis prevalence for all ages and all forms of tuberculosis, with the assumptions that children aged < 15 years were 45.9% of the total population,^10^ and that childhood tuberculosis and extrapulmonary tuberculosis were 10.5% and 6.7% of all total notifications, respectively. This produced a national prevalence for all age groups and all types of tuberculosis of 128 per 100 000 population (95% CI: 94–162). The updated incidence was 175 per 100 000 population (95% CI: 135–215) and the tuberculosis case notification rate was 130 per 100 000 population. Using routine country tuberculosis notification data, the smear positive tuberculosis prevalence, in the population aged < 15 years, was 3 per 100 000, the revised prevalence was 53 per 100 000.

### Performance appraisal

Comparing the estimated case detection rates against global tuberculosis control targets showed that Gambia achieved the 70% case detection target over the period 2009–2013, except for the year 2010 (64.9%; Table 3).

**Table 3:** Tuberculosis case detection rates in Gambia in the years 2009–2013 based on revised tuberculosis incidence estimates

Group	Year				
	2009	2010	2011	2012	2013
Population^a^	1 681 734	1 728 394	1 776 103	1 824 777	1 882 450
Total no. of all types of notified tuberculosis^b^	2 065	1 962	2 249	2 333	2 340
Total no. of estimated incident cases^c^	2 943	3 024	3 108	3 193	3 294
No. of notified cases per 100 000 population	123	114	127	128	124
Case detection rate, % (95% CI)	70.2 (68.5–71.8)	64.9 (63.1–66.6)	72.4 (70.7–73.9)	73.1 (71.5–74.6)	71.0 (69.5–72.6)

CI: confidence interval

^a^ Data source: Gambia 2003 National Census, Central Statistics Department, Government of the Gambia; other years are estimated values.

^b^ Data source: routine reports from the Gambia National Tuberculosis and Leprosy Programme

^c^ Estimated incidence is 175 per 100 000 population.

## Discussion

The overall prevalence of bacteriologically confirmed and smear-positive pulmonary tuberculosis in Gambia were 3.8- and 5.5-fold lower respectively than previously estimated in 2011.^2^^,^^3^ These results are similar to those reported from Ethiopia, where the overall burden of tuberculosis was much lower than previously thought.^11^ Results from other recently concluded surveys in Rwanda, Sudan and Zimbabwe, are also expected to report lower than expected prevalence.^1^ However, recent surveys carried out in other parts of Africa (Ghana,^1^ Malawi,^1^ Nigeria,^12^ the United Republic of Tanzania^1^ and Zambia^13^) revealed higher than expected tuberculosis prevalence. Many of the earlier estimates of tuberculosis burden were derived from mathematical models. Therefore tuberculosis burdens could have been over- or underestimated, as actual measurements now suggest. For Gambia, it is unclear whether earlier figures were exaggerated or understated because of the varying pattern of survey results across Africa. This reinforces the need for reliable country-level measures of tuberculosis burden through surveys such as ours.

The majority of patients with tuberculosis identified in our study were unknown to the national tuberculosis control programme and were not on treatment. While passive case detection has well-known limitations, our data expose the importance of missed diagnostic opportunities within the routine health care system. This is not surprising given the weaknesses of health systems in low-and middle-income countries. While it remains unclear if active case-finding can interrupt tuberculosis transmission in the community through early diagnosis,^14^ strengthening of case-finding through re-training staff and interventions such as the Practical Approach to Lung Health are essential.^15^^,^^16^ Given that tuberculosis prevalence surveys are expensive undertakings (United States dollars 59 per participant in this survey), alternative approaches to active case-finding are required in resource-poor settings. 

Tuberculosis was predominantly found in male participants in our study. The overall male-to-female ratio of 2.0:1 among participants identified with tuberculosis is within the range of ratios reported for Africa and all tuberculosis high-burden countries (0.5:1–3.0:1).^2^ However, this conceals the fact that the male-to-female ratio of 3.7:1 for smear-positive tuberculosis in this survey exceeds the figures of 1.9:1 and 1.5:1 reported for the 22 countries with high-tuberculosis burden and the WHO African Region, respectively. In addition, the male-to-female ratio of 2.2:1 for all participants with bacteriologically confirmed tuberculosis exceeds the 1.7:1 and 1.3:1 reported for the tuberculosis high-burden countries and Africa. This sex difference was consistent across the survey case definitions, despite the lower participation by eligible males.

The majority of identified persons with tuberculosis were in the economically productive 15–44-year age group and this is an important finding for Gambia’s economic well-being. Our survey also showed that tuberculosis prevalence increased with age, and, although the estimates were less precise for the oldest age group, our data points to the need for further investigation of tuberculosis among elderly people in Gambia. While the prevalence of smear-positive tuberculosis did not differ significantly by rural or urban area, urban participants were twice as likely to have bacteriologically confirmed tuberculosis. This suggests increased access to routine and improved tuberculosis diagnostic services especially TB cultures, and particularly in urban areas.

The finding that less than half of all people with tuberculosis would have been identified based on smear microscopy results alone highlights the limitation of this diagnostic approach. Furthermore, the additional number of participants with smear-positive samples identified on the basis of a positive chest X-ray highlights the limitation of a diagnostic algorithm based on a combination of symptoms and smear microscopy alone. Clearly, the use of X-ray in diagnostic algorithms would increase tuberculosis case notifications in Gambia. Our results also demonstrate the added benefit of using additional symptom categories to complement the most common tuberculosis symptom of chronic cough. More than half of participants with identified tuberculosis were smear-negative and culture-positive, indicating that additional diagnostic tools, e.g. rapid diagnostics tests and/or tuberculosis cultures, are needed for early diagnosis.

The clinical epidemiology of nontuberculous mycobacterial disease in most of sub-Saharan Africa and indeed many low and middle-income countries is not well described. In our survey, 5.6% of participants with smear-positive samples were confirmed by culture as due to nontuberculous mycobacteria, pointing to a risk of over-diagnosis of pulmonary tuberculosis under programmatic conditions and the consequent prescription of unnecessary tuberculosis therapy. This further highlights the limitations of smear microscopy as the single diagnostic modality for national tuberculosis programmes in low- and middle-income countries.

The tuberculosis case detection rate was around 70% for the 5 years preceding the survey. Considering the > 85% treatment success already achieved, the Gambia appears to have achieved the DOTS strategy targets for case detection and treatment. In addition, it has achieved the millennium development goal 6c for tuberculosis – to “have halted by 2015 and begun to reverse the incidence of malaria and other major diseases by 2015”^17^ – and the Stop TB Partnership target for halving tuberculosis prevalence; as tuberculosis prevalence has decreased from 350 per 100 000 population in 1990 to 128 per 100 000 in 2013.^18^^,^^19^ There have been substantial investments in tuberculosis control efforts in Gambia, including expansion of access to diagnosis and treatment, and various schemes to provide support to patients, all complemented by advocacy, communication and social mobilization efforts. However, the contribution made by the expanded DOTS programme to improved tuberculosis case detection and notification is unclear. While the DOTS strategy is credited for increasing tuberculosis detection and treatment success and in progress towards achieving global tuberculosis control targets,^20^ other authors argue that it only improves treatment success.^21^ The reduced burden of tuberculosis and the progress made via tuberculosis control efforts in Gambia, support the continued deployment of existing tuberculosis control strategies. However, the results of this survey highlight some gaps that need attention. For example, it is important to elucidate the factors associated with relatively stable tuberculosis notification rates despite achievement of the case detection and treatment success targets in the DOTS strategy.

This survey had some limitations. There were fewer participants than the target sample size but this was mainly the result of lower participation among males than females. Although robust statistical methods were applied to adjust for this, we might not have completely eliminated selection bias and/or the possibility that this survey underestimated the burden of tuberculosis in male participants. Despite the risk of reported sex differences being understated and the less precise estimates for some of the sub-group analyses, we believe the results here are valid because the observed sex differences are consistent with reports from other surveys and the literature. In addition, the survey was not designed to directly measure extrapulmonary or childhood tuberculosis. Although we did not offer voluntary testing and counselling for human immunodeficiency virus infection, such counselling is offered routinely to all newly diagnosed tuberculosis patients at tuberculosis treatment sites in Gambia, and uptake among newly diagnosed persons with tuberculosis exceeds 75%.^1,22^
